# Examination of corneal deposits in nephropathic cystinosis using in vivo confocal microscopy and anterior segment optical coherence tomography: an age-dependent cross sectional study

**DOI:** 10.1186/s12886-020-01336-w

**Published:** 2020-02-26

**Authors:** Anita Csorba, Erika Maka, Otto Alexander Maneschg, Attila Szabó, Nóra Szentmáry, Mária Csidey, Miklós Resch, László Imre, Krisztina Knézy, Zoltán Zsolt Nagy

**Affiliations:** 1 Department of Ophthalmology, Semmelweis University, Budapest, Hungary; 2 1st Department of Pediatrics, Semmelweis University, Budapest, Hungary; 3 Department of Clinical Ophthalmology, Semmelweis University, Budapest, Hungary

**Keywords:** Cornea, Nephropathic cystinosis, Corneal crystals, In vivo confocal microscopy, Anterior segment optical coherence tomography

## Abstract

**Background:**

Presence of corneal cystine crystals is the main ocular manifestation of cystinosis, although controversial findings concerning the corneal layer with the highest density have been reported. The aim of this study was the analysis of the characteristics of crystal arrangement in different corneal layers and the assessment of corneal morphological changes with age.

**Methods:**

A cross sectional study was carried out in three children and three adults who had nephropathic cystinosis and corneal cystine depositions. All patients underwent a comprehensive ophthalmological examination including best corrected distance visual acuity, slit-lamp examination, in vivo confocal microscopy and anterior segment optical coherence tomography. An evaluation of the depth of crystal deposits and crystal density in different corneal layers was also performed. Due to the low number of subjects no statistical comparison was performed.

**Results:**

Anterior segment optical coherence tomography images revealed deposition of hyperreflective crystals from limbus to limbus in each patient. Crystals appeared as randomly oriented hyperreflective, elongated structures on in vivo confocal microscopy images in all corneal layers except the endothelium. In children the deposits occurred predominantly in the anterior stroma, while in adults, the crystals were mostly localized in the posterior corneal stroma with the depth of crystal deposition showing an increasing tendency with age (mean depth of crystal density was 353.17 ± 49.23 μm in children and it was 555.75 ± 25.27 μm in adults). Mean crystal density of the epithelium was 1.47 ± 1.17 (median: 1.5; interquartile range: 0.3–2.4). Mean crystal density of the anterior and posterior stroma of children and adults was 3.37 ± 0.34 (median: 3.4; interquartile range: 3.25–3.55) vs. 1.23 ± 0.23 (median: 1.2; interquartile range: 1.05–1.35) and 0.76 ± 0.49 (median: 0.7; interquartile range: 0.4–1.15) vs. 3.63 ± 0.29 (median: 3.7; interquartile range: 3.45–3.8), respectively. Endothelium had intact structure in all cases. Some hexagonal crystals were observed in two subjects.

**Conclusions:**

In vivo confocal microscopy and anterior segment optical coherence tomography confirmed an age-related pattern of crystal deposition. In children, crystals tend to locate anteriorly, while in adults, deposits are found posteriorly in corneal stroma.

## Background

Cystinosis is a rare autosomal recessive storage disease, which results from mutation in the CTNS gene located at 17p13.2 [1] with an estimated incidence reported to be 1 in 100,000–200,000 live births [1]. CTNS encodes cystinosin, a lysosomal transmembrane protein transporter, which is responsible for transporting cystine amino acid out of the lysosome. Due to the mutation, cystine accumulates in lysosomes and forms insoluble crystals. The crystal deposits appear in various tissues causing functional impairment of the organs [2], especially of the kidneys and of the eyes [3, 4].

Nephropathic or infantile cystinosis is the most frequent (95% of cases) and the most severe phenotype of the disease [5]. It is characterized by renal Fanconi syndrome, which begins in infancy, usually from 6 to 12 months of age [3]. Destruction of kidney tubules leads to end-stage renal failure and usually requires renal transplantation in the first decade of life [2]. The availability of kidney transplantation since the 1960s prolongs life expectancy [3]; however, after transplantation there is an ongoing crystallization and therefore extrarenal manifestations such as ophthalmic complications are more prominent [3, 6].

Cystinosis affects all ocular structures. The most frequently described ocular manifestation is cystine crystal deposition in the cornea [7], but the exact mechanisms of crystal formation are not fully understood yet. In the infantile form the deposits can be seen from the age of 16 months onwards by slit-lamp biomicroscopy [8], and the number of deposits increases with age [9]. Interestingly, different studies have shown contradictory results as to which corneal layer is most affected by crystal deposition [2, 5, 10, 11].

In vivo confocal microscopy (IVCM) is the best imaging technique to represent corneal cystine crystals in vivo [2, 3, 7, 9, 10, 12, 13]. IVCM enables quantification and identification of the deposits at the cellular level and thus this imaging modality is the gold standard for ophthalmological follow-up of patients with cystinosis [1].

Anterior segment optical coherence tomography (AS-OCT) is a relatively novel imaging technique for cross-sectional analysis of the anterior structures of the eye including the cornea. It does not require any contact and hence it is preferable in patients with low compliance for any corneal contact examinations, such as IVCM [7]. Although this examination is faster and less inconvenient for the patients, very few studies are available using AS-OCT for corneal analysis of patients with cystinosis [7, 9].

The literature differs as to which corneal layer has the highest crystal density, and the reason for this difference is unknown. The aim of this study was to investigate whether the age of patients with cystinosis has any influence on corneal crystal morphology and arrangement. In the present work we describe the characteristics of corneal crystals in patients of different ages with nephropathic cystinosis using IVCM and AS-OCT.

## Methods

A cross sectional study was carried out at the Department of Ophthalmology, Semmelweis University with the approval of the Semmelweis University Regional and Institutional Committee of Sciences and Research Ethics. Each participant received information about the study, written consent was obtained from the patients or the parents or legal guardians of subjects under the age of 18 years. All subjects were treated in accordance with the tenets of the Declaration of Helsinki.

Six patients (4 males, 2 females, age 8 years to 36 years) with nephropathic cystinosis were enrolled in our study. Three children (2 boys and 1 girl, aged 8, 12 and 15 years) and three adults (2 men and 1 woman, aged 24, 26 and 36 years) were included. Patients were enrolled if they had previously diagnosed nephropathic cystinosis and if corneal crystals were observed in their eyes by slit-lamp examination. All subjects were receiving oral cysteamine bitartrate (Cystagon®, Orphan Europe S.A.R.L., Puteaux, France) and were on a topical therapy with 0.1% cysteamine hydrochloride (obtained from the University Pharmacy Department of Pharmacy Administration, Budapest, Hungary) applied once to four times daily.

First, patients underwent a comprehensive ophthalmological examination including best corrected distance visual acuity (BCDVA, measured with Snellen chart and converted to logMAR values) assessment followed by slit-lamp biomicroscopy. AS-OCT examinations were performed on all eyes using AngioVue OCT (RTVue-XR, Avanti, Optovue, Fremont, CA, USA), which provides 12 × 9 mm scans with an axial resolution of 5 μm and a transversal resolution of 15 μm. The depth of crystal deposition (DCD) was measured with the caliper function from the anterior corneal surface to the deepest point of crystal deposition in the center of the cornea. Central corneal thickness (CCT) values were measured automatically by the software and derived from the pachymetry map. Finally, IVCM analysis of the central cornea was performed with the Rostock Cornea Module of Heidelberg Retina Tomograph-III (Heidelberg Engineering GmbH, Heidelberg, Germany). Before the examination one drop 0.4% oxybuprocaine (Benoxi®, Unimed Pharma, Bratislava, Slovakia) and one drop of artificial tear gel (0.2% carbomer, Vidisic®, Chem.-pharm. Fabrik GmbH, Brunsbütteler, Berlin, Germany, Bausch&Lomb) were instilled locally in the lower cul-de-sac. The microscope lens was covered by a sterile polymethyl methacrylate cap (TomoCap®, Heidelberg Engineering GmbH). Images were taken of each layer in the central cornea from the epithelium to the endothelium using the instrument’s section mode. IVCM images represent an en face section of the cornea with a resolution of 384 × 384 pixels covering a 0.4 × 0.4 mm^2^ area. No corneal complications developed due to the IVCM examination. All IVCM measurements were recorded, and images were analyzed by the same examiner (A.Cs.). The crystal density (CD) was quantified using CD scoring system as previously published [9]. CD was evaluated in different corneal layers, i.e. the epithelium, anterior stroma, posterior stroma and endothelium (graded from 0 to 4: 0, no crystal; 1, < 25%; 2, 25 to 50%; 3, 50 to 75%; 4, > 75%; the percentage was based on the quotient of the area affected by crystal deposition and the whole field of each image). The CD of one corneal layer represented the mean of 5 pictures analyzed. The values of DCD and CD for patients of different ages were evaluated. No statistical analysis was performed due to the low number of patients.

## Results

A total of 12 eyes of 6 patients with nephropathic cystinosis were examined in our study. All subjects were in good general health. The three adults and the oldest child underwent renal transplantation previously. The involved patients under 18 years old were siblings. The majority of subjects had normal visual acuity. Slit-lamp biomicroscopy revealed diffuse crystal deposition throughout the entire cornea in all patients. In Patient 1–5 there was not detectable any sign of corneal oedema or scarring. As a late-onset complication of corneal involvement of nephropathic cystinosis Patient 6 had corneal scarring on both of her eyes and a plaque-like band keratopathy on the left cornea, which explains her low visual acuity and high corneal thickness (674 μm). The mean CCT in the remained 11 eyes of 6 examined patients was 582.45 ± 25.91 μm. Clinical characteristics of the patients are shown in Table 1.

**Table 1:** Clinical characteristics. BCDVA = best corrected distance visual acuity (logMAR)

Patient	Age (years)	Gender	Renal transplantation	Eye	BCDVA
1	8	male	no	OD	0.0
				OS	0.0
2	12	male	no	OD	0.0
				OS	0.0
3	15	female	yes	OD	0.0
				OS	0.0
4	24	male	yes	OD	0.0
				OS	0.0
5	26	male	yes	OD	0.0
				OS	0.0
6	36	female	yes	OD	1.0
				OS	1.0

**Fig. 1:**
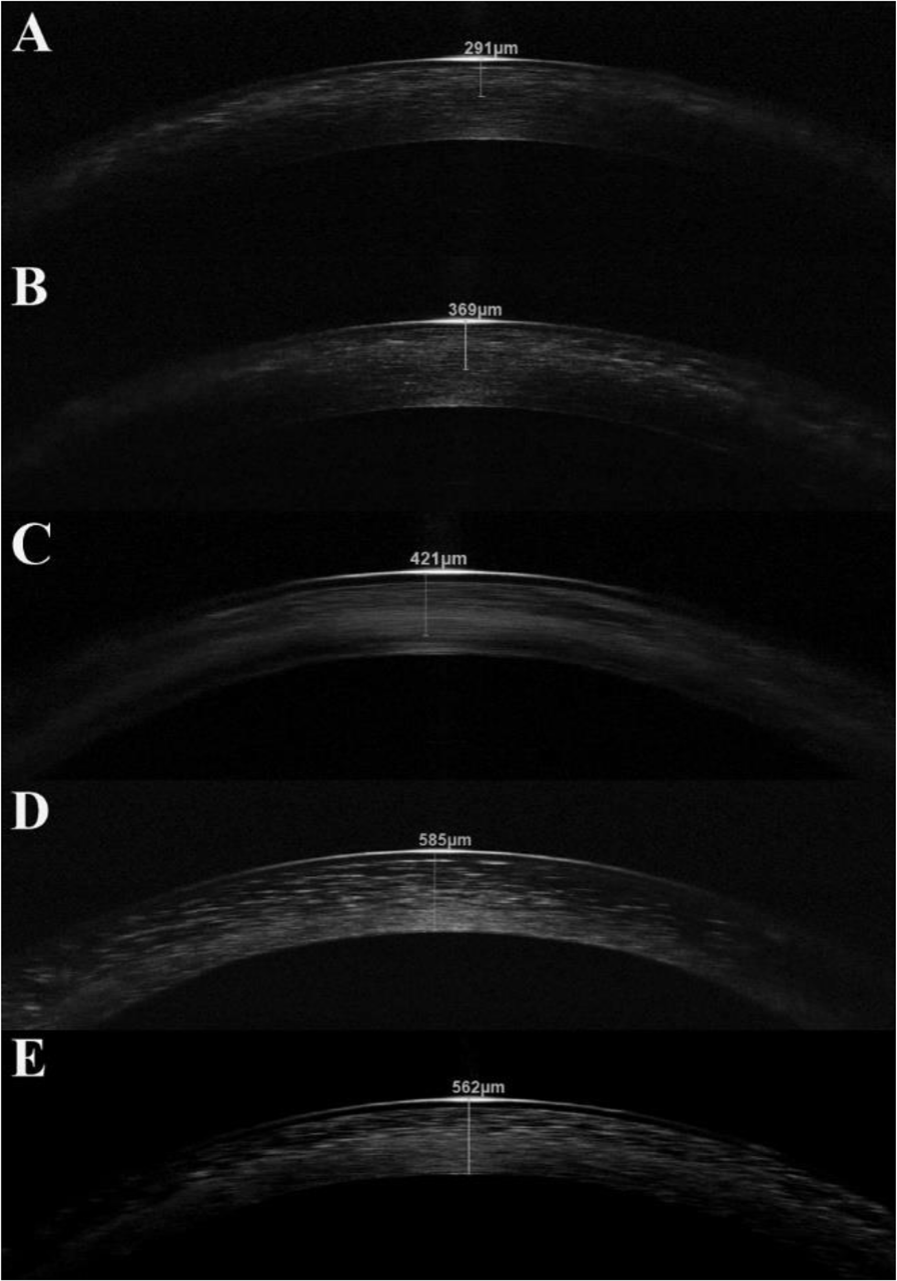
Anterior segment optical coherence tomography (AS-OCT) scans of the cornea in nephropathic cystinosis in ascending order with age. (**a**–**c**) In children, the anterior stroma is predominantly involved in crystal deposition. In Patient 3 (C) the crystal location is deeper, but the posterior stroma is still not affected. (**d**–**e**) On the contrary, in adults the crystals are mostly located in the posterior stroma

AS-OCT images revealed deposition of hyperreflective crystals from limbus to limbus in each patient. Patient 6 was excluded from DCD measurement because due to the corneal scarring appearing as a hyperreflective area on AS-OCT scans, exact distinguishment of hyperreflective crystals from scarred areas was not possible. The scans showed that with increasing age the crystal deposits are located deeper (Fig. 1a–e). In younger children only the anterior stroma is affected (Fig. 1a, b), whereas in the oldest child (15 years old) the majority of the deposits affects the anterior and the middle stroma as well (Fig. 1c). In these patients, crystals in the posterior parts were not observed. In adults, the crystals are mostly localized in the posterior two-third of the corneal stroma (Fig. 1d,e).

**Fig. 2:**
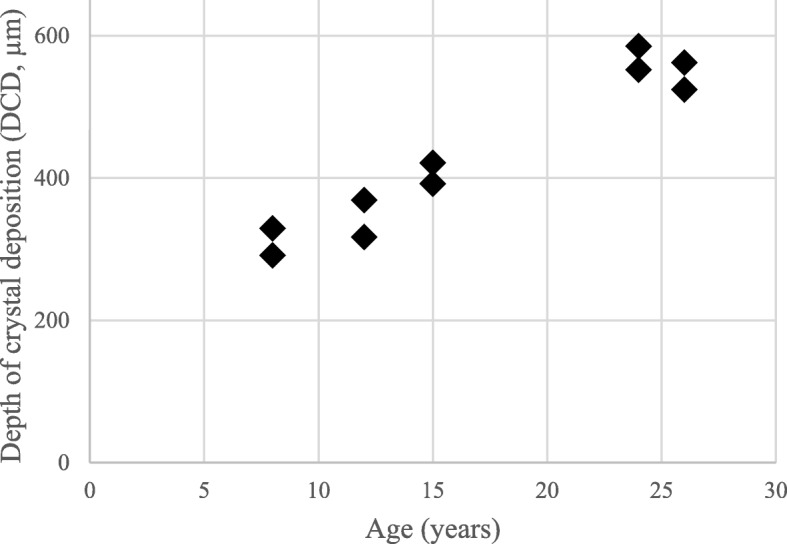
Depth of crystal deposition (DCD) measured on anterior segment optical coherence tomography images. DCD values show an increasing tendency with age. In all patients available for the measurement the DCD values were symmetrical in both eyes that is observable on the distribution of the plot

The mean DCD was 353.17 ± 49.23 μm in children and it was 555.75 ± 25.27 μm in adults. Figure 2 shows the DCD values plotted against the age of the subjects. On the graph, the two eyes are showing similar values, and age-related tendency of crystal deposition can be seen.

**Fig. 3:**
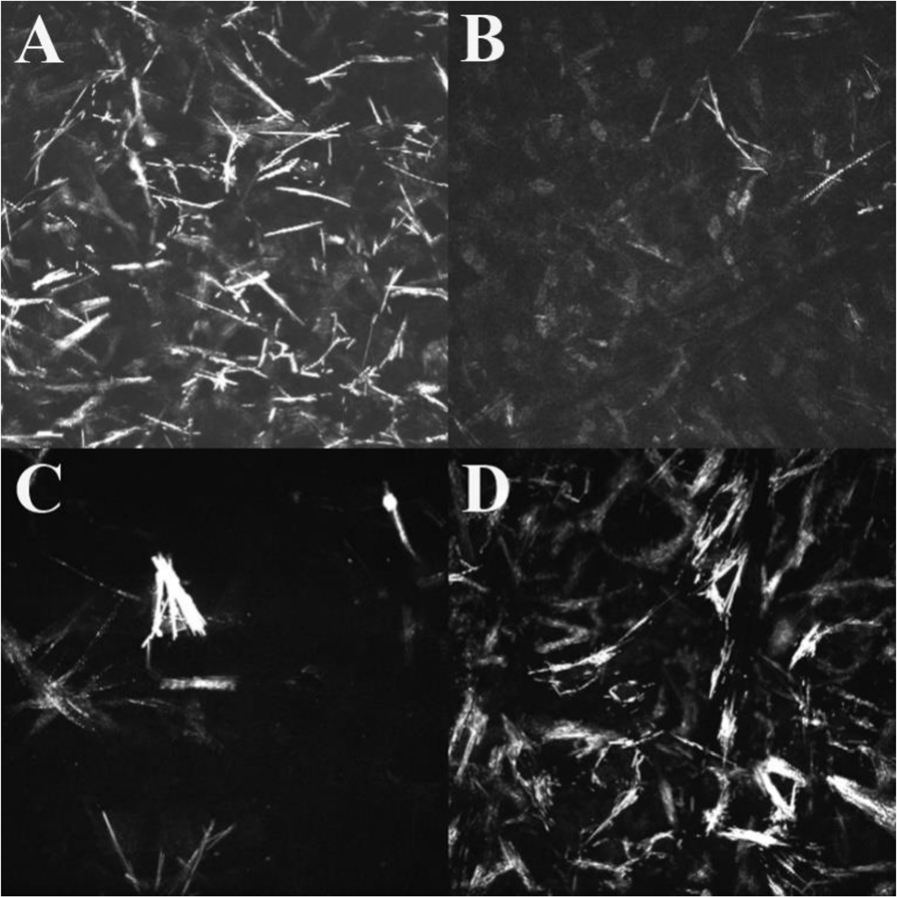
In vivo confocal microscopy (IVCM) images showing different stromal arrangement of the deposits. (**a**) In children, many deposits are in the anterior stroma. (**b**) The posterior stroma is intact in children: few crystals and many keratocytes are visible. (**c**) On the contrary, few crystals are aggregated in the anterior stroma of adults, whereas (**d**) their posterior stroma has high crystal density

**Fig. 4:**
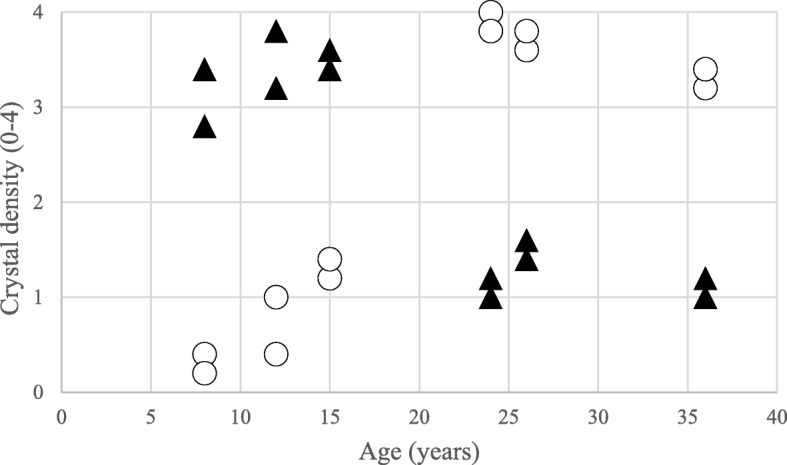
Crystal density (CD) of anterior and posterior stroma. Semiquantitative analysis of deposits showed higher density in the anterior stroma in children and higher density in the posterior stroma in adults. ▲ CD in the anterior stroma; **○** CD in the posterior stroma

On IVCM images the crystals appeared as hyperreflective, sharply demarcated, elongated structures in different size and shape. In each layer, the crystals were oriented randomly without any differentiation and could be observed throughout the entire the cornea except the endothelium. Within the epithelial layer short and thin deposits were seen extracellularly in nine eyes of five patients. The mean CD of the epithelium was 1.47 ± 1.17 (median: 1.5; interquartile range [IQR]: 0.3–2.4). All examined patients had crystals in the full thickness of corneal stroma. In our three children, narrow and needle-shaped deposits were found predominantly in the anterior stroma. On these images keratocytes were not detected (Fig. 3a). The mean CD in the anterior stroma of children was 3.37 ± 0.34 (median: 3.4; IQR: 3.25–3.55). In adults thick and long crystals were aggregated in the anterior stroma and they were surrounded by homogeneous, acellular hyporeflective areas (Fig. 3c). The mean CD in the anterior stroma of adults was 1.23 ± 0.23 (median: 1.2; IQR: 1.05–1.35). In the anterior stroma CD was found to be greater in younger patients than in older ones (Fig. 4). In the posterior corneal stroma of children crystals were narrower, keratocyte nuclei were seen clearly, and normal stromal structure was detected with a mean CD of 0.76 ± 0.49 (median: 0.7; IQR: 0.4–1.15) (Fig. 3b). The cell borders of keratocytes were not visible; therefore, it could not be determined whether the crystals were located intra- or extracellularly. In adults, the highest crystal density was found in the posterior stroma with a mean CD of 3.63 ± 0.29 (median: 3.7; IQR: 3.45–3.8). Other cells could not be detected in this layer, either (Fig. 3d) (Fig. 4). The endothelial cell layer had an intact structure in all cases. Data of DCD and CD values in different corneal layers are shown in Table 2.

**Table 2:** Central corneal thickness (CCT) in μm, depth of crystal deposition (DCD) in μm and crystal density (CD) values in the study subjects. The CD values were derived from five measurements in the given area

Patient	Eye	CCT	DCD	CD			
				Epithelium	Anterior stroma	Posterior stroma	Endothelium
1	OD	613	329	1.6	3.4	0.4	0.0
	OS	627	291	1.2	2.8	0.2	0.0
2	OD	611	317	1.4	3.2	1.0	0.0
	OS	583	369	2.4	3.8	0.4	0.0
3	OD	582	392	3.2	3.4	1.2	0.0
	OS	550	421	2.4	3.6	1.4	0.0
4	OD	564	552	0.0	1.2	4.0	0.0
	OS	554	585	0.4	1.0	3.8	0.0
5	OD	555	562	2.0	1.4	3.6	0.0
	OS	587	524	3.0	1.6	3.8	0.0
6	OD	581	–	0.0	1.0	3.2	0.0
	OS	674	–	0.0	1.2	3.4	0.0

**Fig. 5:**
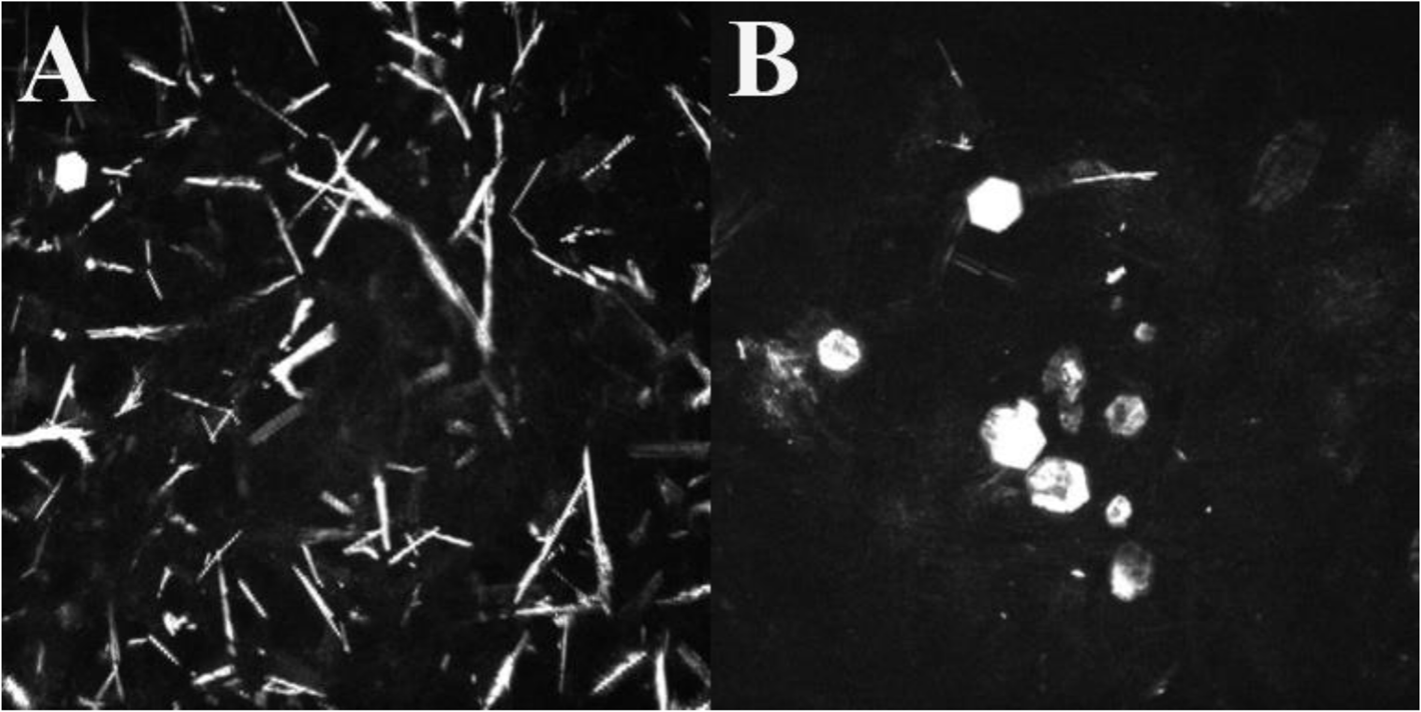
Hexagonal crystals. (**a**) One hexagonal crystal is visible on the IVCM image of a child. (**b**) Some hexagonal crystals in adult

According to the IVCM recordings, most of the corneal crystals were oblong and needle-shaped. Interestingly, hexagonal crystals were also detectable in two patients. In Patient 1 (8 years old) only a few of these crystals were visible in the anterior stroma (Fig. 5a). In Patient 5 (26 years old), several hexagonal crystals were detected in both the anterior and posterior stroma (Fig. 5b).

## Discussion

To the best of our knowledge, this is the first study to investigate the properties of crystal localization in cystinosis in different age groups, by employing IVCM and AS-OCT imaging. In our report 12 eyes of 6 patients with nephropathic cystinosis were assessed to describe the appearance of corneal cysteine crystals, which is a characteristic symptom of this disorder [3]. Epithelial involvement was observed in 5 out of 6 patients, in agreement with the results of several previous studies [2, 7, 9, 14]. Interestingly, the endothelium was spared in all cases in this series, although some previous data indicate that endothelial involvement may also occur [11, 15].

In the literature there are contradictory data on the stromal pattern of corneal crystal deposition. Most of the studies describe that the majority of the crystals is located in the anterior or in the posterior stroma [9, 10], while some case reports describe the main involvement of the middle layer [2, 5]. Previous histopathological and clinical studies confirmed that crystal deposition begins at the anterior and peripheral part of the cornea and spreads posteriorly and centrally with aging [11, 16, 17]. Indeed, our results were in accordance with these previous reports: in our three children, crystal deposition mostly affected the anterior stroma, while in adults, the posterior stroma had the highest crystal density. We also found that despite this localization trend the entire corneal stroma was affected in all ages. According to our data, crystals appear throughout the whole thickness of the cornea with older age. Both AS-OCT and IVCM showed that accumulation of the crystal deposits has increasing tendency in the posterior stroma with aging.

Our results may suggest that the deposition of cystine crystals is associated with changes in keratocyte population. In regions where crystals were deposited, keratocyte dropout was observed. Hence, in children, the posterior stromal parts had normal keratocytes, whereas in adults, keratocytes could not be observed at full thickness. In vitro studies reported progressive keratocyte disruption, keratocyte loss in corneal cystinosis and higher apoptotic rate in nephropathic cystinotic fibroblasts [18, 19]. These results support our in vivo findings about the absence of keratocytes in areas affected by crystal deposits.

Moreover, hexagonal crystals were found in two patients located in the stroma. According to histopathological and IVCM studies, corneal crystals are usually needle-shaped [10, 11, 20, 21], while they have hexagonal or polygonal configurations in the other parts of the body [22]. Dixon et al. emphasized the role of the stromal collagen in needle-shaped crystallization [23]. According to their results, due to the charge-charge interactions between the anionic collagen fibrills and cationic cystine, cystine amino acid is bound to the surface of collagen. The tightly located collagen lamellae inhibit the three dimensional growth, hence the polygonal forming of cysteine crystals [23]. Presentation of hexagonal deposits in our patients suggests that the regular, tightly packed structure of the collagen lamellae in the cornea may change, thus, the growth of the crystals may occur not only parallel, but in vector direction of the collagen fibers, as well.

In nephropathic cystinosis several age-related corneal complications have been reported i.e. band keratopathy, corneal neovascularization, filamentary keratopathy and corneal scarring [24, 25], but the exact cause of these complications remained unclear. So far, we are not aware of any association between the changes in stromal microarchitecture and the risk of developing any late-onset corneal complication in nephropathic cystinosis. Future prospective studies are required to elucidate the significance of crystal location and stromal alterations in the development of late-onset complications.

We performed AS-OCT examinations in all our patients. Due to the non-invasive nature of AS-OCT, examination can be performed easily even in very young patients (see the 8-year-old subject in our study). On cross-sectional scans it is easy to determine which part of the cornea has the highest density of crystals; however, epithelial and endothelial involvement could not be determined by this imaging method due to its lower resolution. Moreover, corneal scarring makes impossible exact DCD value measuring, because crystals are not visible in the area of the scar.

Our study suggests that due to its higher magnification (approximately 800×) and higher resolution (1–2 μm/pixel), IVCM could be more advantageous than AS-OCT for the assessment of corneal deposits in cystinosis. First, with this method we were able to evaluate the involvement of epithelium and endothelium. Second, the deposits could be observed in regions of corneal scarring as well, and their amount could be determined. Finally, we found deposits throughout the full corneal thickness in all examined patients, even in those patients who had only anterior corneal involvement according to DCD values on AS-OCT images. Although IVCM is able to assess corneal involvement very precisely, this technique cannot be performed on patients who have difficulties with contact examination. The severity of corneal involvement has been assessed in previous studies using the Gahl-score, based on a collection of slit-lamp photographs of corneas containing cystine crystals at different densities [8]. In 2009, Labbé et al. published a new IVCM scoring system which provides more accurate evaluation of crystal density than the aforementioned Gahl-score [9]. Another advantage of the IVCM scoring system is that it enables the comparative evaluation of crystal density in different layers of the cornea.

Precise location and depth of crystals within corneal layers can only be evaluated by IVCM and AS-OCT. However, in most of clinical studies investigating the effects of topical cysteamine hydrochlorid (CH), crystal density was evaluated according to slit-lamp photography [8, 14, 26–29]. No study is currently available about life-long effects of topical CH drops on exact arrangement of deposits and about its impact on age-dependent changes. Diffusion of hydrophilic CH through the lipophilic epithelium is low [30]. Due to short corneal contact time, dose regimen is frequent up to 12 times per day and the aqueous solution of CH is unstable at room temperature [31]. These factors impose a significant burden on patients and may lead to poor adherence [31]. Efficacy of 0.1% CH drops in reduction of crystal density is low even in anterior stroma [12]. All these limitations make it less likely that topical CH would have profound impact on the arrangement of deposits at full depth of the cornea. Recently, a new gel solution of CH has been developed. High viscosity of the gel form prolongs corneal contact time, which contributes to deeper penetration and less frequent administration (3–5 times per day) [12, 13].

In this manuscript, we analyzed the properties of cystine crystal location and the morphology of corneal structure in different ages. The rare occurrence of the disease could not make it possible to involve more subjects, so the potential limitation in our study could be the low number of cases. However, only case reports [2, 5, 10] and one research article [9] with low number of subjects can be found using IVCM and/or AS-OCT in which crystal localization is also reported. Thus, we believe taking into consideration this limitation our study provides important results and shows clearly the inherent tendency of linearity between the age and stromal density of the deposited crystals. The other main limitation of our study is the lack of statistical evaluation. We have attempted to divide the patients into two subgroups to represent the differences between younger and older age, however, we could not statistically evaluate the average values of these two groups because of the small number of patients. The present study was not primarily concerned with the distinction between children and adults in terms of deposition of crystals, but showed the relationship between localization of the crystals and the age of the patients. Due to the rarity of cystinosis, recruiting a large cohort of these patient in different ages is challenging, but it could provide precise statistical evaluation which may confirm our results.

## Conclusion

In our study, we provided new data about corneal microarchitecture in nephropathic cystinosis. Both AS-OCT and IVCM are seemingly useful methods to assess the cystine crystals in the cornea in cystinosis and desirable to be used for detection of morphological features of cystine crystals and corneal alterations. Our results suggest that there is a tendency of age-related redistribution of crystal deposits in the posterior stroma, while loss of keratocytes can be observed. Some hexagonal crystals may occur in the stroma, but the exact cellular mechanism is unknown. These results could be useful to explore morphological changes of the cornea in nephropathic cystinosis. Nevertheless, further prospective studies, also with in vitro examinations, are required to elucidate the process of corneal changes and exact cellular and molecular pathomechanisms in nephropathic cystinosis with aging.

## Data Availability

The datasets used and/or analyzed during the current study are available from the corresponding author on reasonable request.

## Abbreviations

AS-OCT: anterior segment optical coherence tomography
BCDVA: best corrected distance visual acuity
CCT: central corneal thickness
CD: crystal density
CH: cysteamine hydrochlorid
DCD: depth of crystal deposition
IQR: interquartile range
IVCM: in vivo confocal microscopy
